# Development of equipment that promotes exercise training for children with orthostatic intolerance

**DOI:** 10.3389/fped.2025.1577253

**Published:** 2025-04-09

**Authors:** Yuko Ishizaki, Yoshitoki Yanagimoto, Asahi Yoshida, Kiyoshi Hayakawa, Kazunari Kaneko

**Affiliations:** ^1^ Department of Pediatrics, Kansai Medical University, Osaka, Japan; ^2^ Department of Industrial Systems Engineering, Osaka Metropolitan University College of Technology, Osaka, Japan

**Keywords:** orthostatic intolerance, child, exercise training, deconditioning, monitoring

## 1 Introduction

Orthostatic intolerance (OI) is common in pediatric practice, and children with OI have difficulty enduring an upright posture (1). Being a syndrome, the main pathologies of OI include orthostatic hypotension (OH), postural orthostatic tachycardia syndrome (POTS), and postural syncope (2). Healthy children can rise from the supine position and perform their daily activities, whereas children with OI exhibit various symptoms when moving from the supine position to an upright position, such as dizziness, lightheadedness, headache, palpitations, and fatigue, which are caused by cerebral hypoperfusion (1–4). The upright posture stresses regulatory capabilities and causes venous pooling (2). Muscle pumps usually push blood back to the heart when upright and during exercise, enabling the skeletal muscle pump to form an important class of physical countermeasures against OI (5). Conversely, if muscle pump function is impaired, OI symptoms will worsen.

Cardiovascular deconditioning due to physical inactivity has been reported to cause OI in healthy young individuals (6, 7). Gravitational deconditioning encompasses a markedly reduced blood volume, blood volume redistribution, trophic cardiac atrophy, baroreflex changes, diminished vasoconstriction to pressor drugs, skeletal muscle atrophy resulting in the loss of the skeletal muscle pump, and osteoporosis (1, 8). In patients confined to bed, OI may take the form of POTS, OH, or vasovagal syncope, and bed rest worsens these states if they are already present (1, 2). Deconditioning was observed in healthy children during the COVID-19 pandemic lockdown (9). Physical inactivity can either worsen existing OI symptoms or result in OI through deconditioning. From this perspective, physical exercise is one way to improve the chronic symptoms of OI.

The expert consensus on POTS states that it is essentially managed by nonpharmacological therapy, such as fluid intake and aerobic exercise, which are more effective than pharmacological treatment in adult patients (10). Exercise is considered an essential treatment for both OH and POTS (2). However, because children suffering from severe OI have deteriorated exercise tolerance, exercise must be started at a mild intensity level and then increased gradually.

Through this narrative review, we found the four key points for effective exercise training for children with OI: exercise at approximately 70% of maximum oxygen intake using heart rate (HR) as an indicator; exercise should be started in a supine or semi-supine position; it should consist of a warm-up, target exercise, and cool down; and as exercise tolerance improves, the exercise intensity and duration should also increase (11). Non-experts in exercise therapy may find it challenging to provide appropriate exercise for children with OI; therefore, training methods and equipment for those with a high evidence level are strongly needed.

To promote appropriate exercise training for children with OI, we are developing an ergometer with a monitor that will display the appropriate exercise training that children with severe OI can perform in the recumbent position.

## 2 Case presentation of a boy with severe OI who underwent recumbent exercise training

### 2.1 Case presentation

The patient was a 13-year-old boy, first-year junior high school student. He entered junior high school in April. Three months later, in July, he began to experience stomachache, vomiting, and headaches. After the summer vacation, he began to wake up feeling uncomfortable. He was a member of the basketball club activity; however, after the summer vacation, he was unable to go to school and attend the basketball club. He stayed lying down all day in his home. Subsequently, he was diagnosed with OI at a hospital and was started on pharmacotherapy, which was ineffective. His condition worsened, even short walks caused fatigue, and he became housebound. Six months later, he was referred to our hospital for specialized training for OI. On the first visit to our hospital, he complained of severe general fatigue and dizziness with slight movements. He was instructed to drink fluids and exercise; however, he complained that even short walks were tiring. He became aware of his declining physical strength. He therefore requested to undergo bed ergometer exercise training.

### 2.2 Exercise training regimen

The exercise regimens were based on several reviews and guidelines (1, 11–13) and should be performed once a day for 30 min for 5 days a week.

Cardiopulmonary exercise testing (CPX) is performed to assess the exercise tolerance of patients before and after the exercise training. CPX includes the measurement of respiratory gas and is the gold standard for evaluating aerobic fitness and examining integrated physiological responses to exercise in pediatric medicine (14). CPX is usually used for the evaluation of adult patients with cardiac or respiratory diseases (15), and an exercise prescription is one of the potential indications (15). Children with chronic conditions often limit their participation in physical activities, which leads to deconditioning, reduced functional ability, and downward spiral of further hypoactivity (14). It is also a possible indication of exercise training assessed by CPX. The measurement of the maximal oxygen uptake (VO_2_) or peak VO_2_ during a progressive CPX up to maximal exertion is widely considered the gold standard for assessing aerobic fitness (14). For exercise prescription, the knowledge of anaerobic threshold (AT) can also be useful (15), so we used HR, peak VO_2_, and AT VO_2_.

One approach aims for 60%–70% of a patient's HR achieved during maximal exercise testing beginning with semirecumbent exercise. This involves 5 min of warm-up to achieve the target, 15 min at the target, and then 5 min of cooling down (1). As exercise tolerance improves, 5 min is added per session at the target until one can complete 30 min at the target HR (1). Our exercise regimen was modified to their one with the patient in a recumbent position, i.e., the spine position, rather than a semirecumbent position at the beginning of the exercise training, and then after 2 weeks, when the patient's exercise tolerance had improved, the bed was raised to a semirecumbent position, and the exercise load was increased.^1^

### 2.3 Monitoring of exercise with sensors on the pedals

The combination of frequency, intensity, and duration of chronic exercises was found to be effective in triggering a training effect (14). It should be helpful to perform exercises to strengthen endurance at a constant speed, and this helps visualize the appropriateness of the exercise. Then, a monitor on the pedals of the recumbent ergometer used in the exercise training was set for this case. This monitor measured the pressure with which the patient pressed the pedals and the speed at which they pedaled. This monitor used Bluetooth and could be monitored from a PC located away from the bed (16).

**Figure 1:**
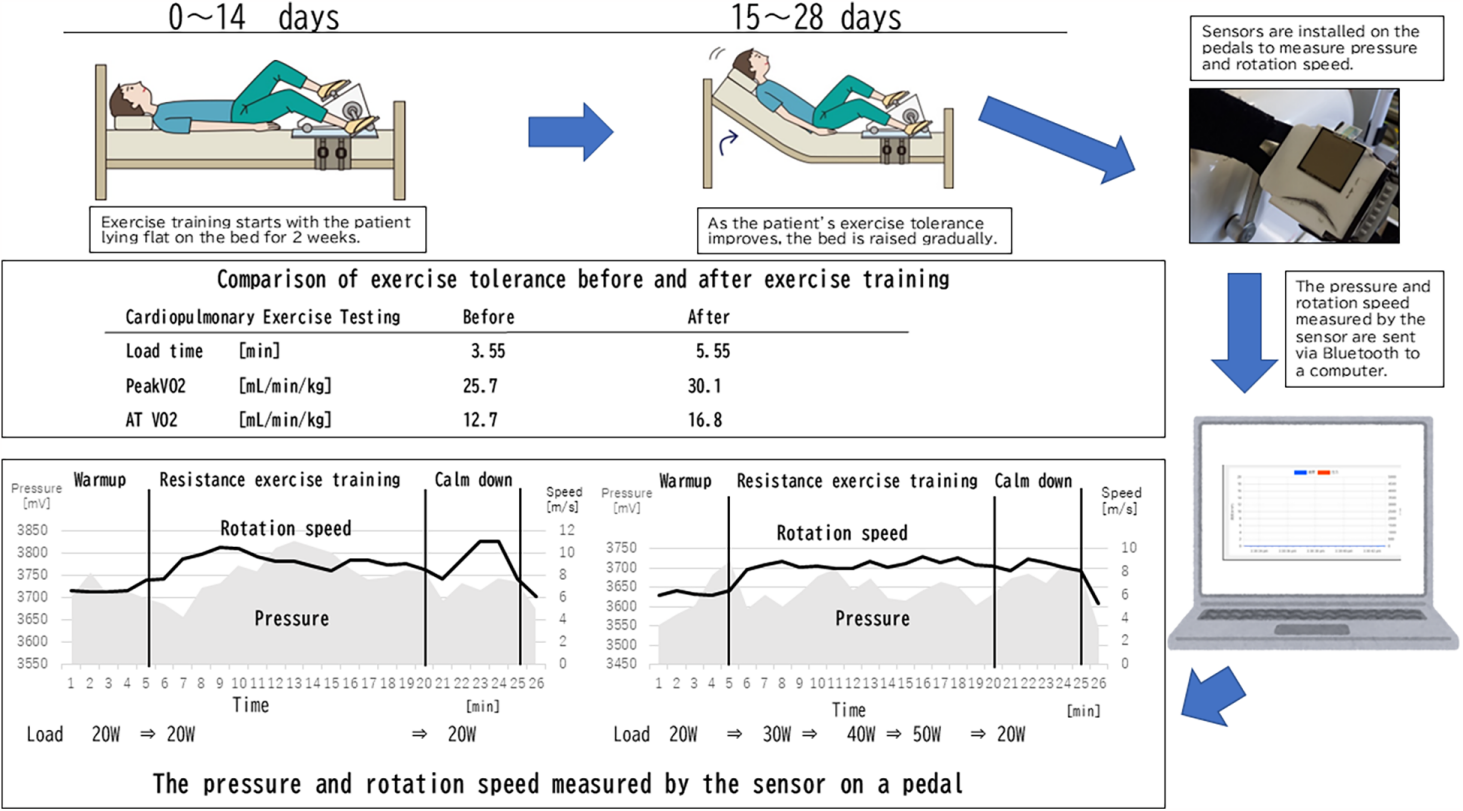
Exercise training, cardiopulmonary exercise testing, and monitoring diagram. The pressure value is the voltage converted into a number, and the actual pressure *P* [N] can be expressed by the following formula, assuming the voltage on the graph is V [mV]: $P=20\log10^{\frac{V}{4096}}$.

During monitoring on the first day of exercise, the pedaling speed was unstable, even though the exercise load was kept at 20 watts (W). After the first day of exercise therapy, he complained of muscle pain and severe fatigue. By week 4, he could perform ergometer exercises with his upper body almost entirely raised in bed, and the load had been increased to 50 W. The pedaling speed remained constant even when the load was increased, and he was able to perform a stable exercise (Figure 1).

### 2.4 Evaluation of the effectiveness of exercise training

The exercise program calculated from the CPX results was a target HR of 100/m and an exercise load intensity of 46 W. The actual exercise loads were a maximum load of 30, 40, and 50 W in weeks 1, 2, and 3 and 4. After 4 weeks, the exercise training was completed without any accidents.

The CPX results before and after the exercise training are shown in Figure 1. At the beginning, the patient had low exercise tolerance compared with his peers and had improved after 4 weeks of training. Various indices have improved as follows: load time, which is possible exercise duration (min), at 3.55–5.55, peak VO_2_ (ml/min/kg), of 25.7–30.1, and AT VO_2_ (ml/min/kg) of 12.7–16.8, respectively. Before the exercise training, the patient experienced fatigue and had been confined to his home. After the training, he became confident in his physical strength and could engage in the sports activity without complaining OI symptoms.

The summary of the standing test before and after exercise training is as follows: (1) At the beginning of the exercise training, the patient complained of fatigue and stopped halfway through the 10-min standing test. However, after 4 weeks of training, the patient was able to complete the entire test; (2) The maximum heart rate at the beginning was 123 (beats/min), at which point the test was stopped. After exercise training, the maximum heart rate increased to 142 (beats/min); (3) The change in pulse pressure was 56 (beats/min) to 47 (beats/min); (4) No decrease in blood pressure was observed in any of the tests.

The results of the standing test suggest an improvement in orthostatic tolerance, as the patient was able to complete the 10-min standing test after training. Additionally, the small change in pulse pressure during the standing test likely indicates improved circulation while standing.

## 3 Perspective

To manage the exercise training of children with severe OI, sensors were installed on the pedals to show the pressure and speed that demonstrate appropriate training. Exercise must be continued at a constant pressure and at a speed to improve endurance. When performing the ergometer exercise on a bed, children tend to pedal faster and gain momentum or stop exercising without supervision. However, it is not easy for experts to be with the children every day and supervise their exercise. Thus, we developed this sensing system. We separately reported on the effectiveness of exercise training for children with OI (see text footnote 1), and in conducting the study, a specialist for exercise training accompanied the children during their exercise for 30 min every day. However, this method cannot be widely disseminated. Therefore, convenient tools that will not only prove the effectiveness of exercise therapy for children with OI but also enable exercise therapy in medical institutions without specialists must be developed. If exercise training can be implemented in ordinary homes, the deterioration of OI due to deconditioning can be prevented, which improves the quality of life of children with OI.

## Footnotes

^1^Yanagimoto Y, Ishizaki Y, Yoshida R, Terashima T, Ishitani K, Haraguchi K, et al. Evaluation of effectiveness of exercise training therapy for circulatory dynamics of children with orthostatic dysregulation. *Front Pediatr*.
